# Fabrication of Gastro-Floating Famotidine Tablets: Hydroxypropyl Methylcellulose-Based Semisolid Extrusion 3D Printing

**DOI:** 10.3390/pharmaceutics15020316

**Published:** 2023-01-18

**Authors:** Hyun Seok Yang, Dong Wuk Kim

**Affiliations:** BK21 FOUR Community-Based Intelligent Novel Drug Discovery Education Unit, Vessel-Organ Interaction Research Center (VOICE, MRC), Research Institute of Pharmaceutical Sciences, College of Pharmacy, Kyungpook National University, Daegu 41566, Republic of Korea

**Keywords:** hydroxypropyl methylcellulose, famotidine, semisolid extrusion 3D printing, gastroretentive floating system, dissolution kinetics

## Abstract

Semisolid extrusion (SSE) three-dimensional (3D) printing uses drug-loaded paste for the printing process, which is capable of constructing intricate 3D structures. This research presents a unique method for fabricating gastro-floating tablets (GFT) using SSE. Paste-loaded famotidine with a matrix made of hydroxypropyl methylcellulose (HPMC) were prepared. Nine 3D printed tablets were developed with different HPMC concentrations and infill percentages and evaluated to determine their physicochemical properties, content uniformity, dissolution, and floating duration. The crystallinity of the drug remained unchanged throughout the process. Dissolution profiles demonstrated the correlation between the HPMC concentration/infill percentage and drug release behavior over 10 h. All the fabricated GFTs could float for 10 h and the Korsmeyer-Peppas model described the dissolution kinetics as combination of non-Fickian or anomalous transport mechanisms. The results of this study provided insight into the predictability of SSE 3D printability, which uses hydro-alcoholic gel-API blend materials for GFTs by controlling traditional pharmaceutical excipients and infill percentages. SSE 3D printing could be an effective blueprint for producing controlled-release GFTs, with the additional benefits of simplicity and versatility over conventional methods.

## 1. Introduction

Three-dimensional (3D) printing is the process of using a printer to create 3D products rather than flat text or images. Three-dimensional printing technology can manufacture products on-site in real-time by layering specific materials [1]. The applications of 3D printing are sufficiently numerous enough to include cases in almost every field, including architecture, aerospace, domestic product, pharmaceutical, and medical fields [2,3,4,5,6]. As such, 3D printing technology is one of the core technological fields leading the fourth industrial revolution due to its versatility that can be used in various fields.

Meanwhile, there are various applications for 3D printing in the pharmaceutical industry. It is possible to use customized medicine fabrication to manage the drug dosage required for the patient [7], manufacture a little amount, and develop a dosage form with a complicated internal structure and novel shape (hollow shape, porosity, and drug compartmentalization, etc.) and this allows for drug release control, such as immediate release or sustained release [8]. SPRITAM^®^, the first medication created using 3D printing, was approved by the US Food and Drug Administration in August 2015 [9]. SPRITAM^®^ is a fast-disintegrating formulation with a porous structure manufactured using Zipdose technology, which is a 3D inkjet printing method for rapid disintegration, instead of the traditional tablet manufacturing process. Its porous structure enables rapid disintegration with only a small amount of water in the mouth, which can help patients with difficulties swallowing tablets. With the development of SPRITAM^®^, it has been confirmed that manufacturing using 3D printing technique is feasible, presenting a novel approach to the traditional pharmaceutical production methods that have been used for over 100 years.

In addition to the previously mentioned inkjet method, various 3D printing methods have been developed, such as material extrusion, selective laser sintering, and vat photopolymerization [10]. Among these technologies, recent research has focused on material extrusion printing techniques employing fused deposition modeling (FDM) and semisolid extrusion (SSE) techniques in the pharmaceutical industry. Hot-melt extrusion produces the starting material for FDM by combining active pharmaceutical ingredients with thermoplastic polymers (PVA) [11,12] to create long cylindrical, rod-shaped filaments. These filaments are fed into the heating nozzle, melted, and deposited layer-by-layer with FDM to produce the desired 3D products. It has the advantage of being simple to use and inexpensive compared to other types of printers, but it has the limitation of not being able to use drugs and excipients that are not heat-stable because it requires a filament production and printing process that increase the temperature [13].

To overcome this limitation, the SSE printing technique is used. Compared to FDM technology, SSE printing does not require high-temperature conditions for operation [14]. As a result, it is used in biological applications, tissue engineering, and food processing requiring temperature-sensitive substances such as living cells [15], protein enzymes, probiotics [4], vitamins [16], and enzymes [17]. In the field of pharmaceutical application, SSE 3D printing was firstly used to produce controlled release bilayer tablets [18] or polypills [17]. Ever since, SSE technology has rapidly evolved to manufacture other types of dosage forms, from gastro-floating tablets [19], chewable tablets [20] and orodispersible films [21], to rectal suppositories [22], implantable patches [23], and high drug loading tablets [24]. The main advantage of SSE 3D printing is the relatively low operating temperature, the simplicity of the process, and the large number of available excipients [25]. Based on this, SSE 3D printing is expected to be used more actively in the future for the fabrication of pharmaceuticals.

The gastro-retentive drug delivery method prolongs the gastric retention time and delays drug release compared to traditional dosage forms—allowing the drug concentration in plasma to remain constant without rapidly changing—and reduced frequent drug administration, reducing side effects to improve patient compliance [26]. Furthermore, it has the advantage of enhanced bioavailability. Methods to increase gastric residence time include floating [27,28], expandable [29], high-density systems [30], and bio-adhesive systems [31]. The gastro-floating system is a promising method for prolonging intragastric residence time and sustained-release patterns among various gastro-retentive systems. At the same time, unlike other methods, this method is not influenced by peristalsis in the gastrointestinal tract. The two most commonly used methods are the effervescent and non-effervescent systems [32]. The effervescent system generates buoyancy in CO_2_ gas by chemically reacting NaHCO_3_, MgCO_3_, CaCo_3_, or other substances with water. The gases generated during this process can cause gastrointestinal problems and unwanted side effects [33]. In contrast, the non-effervescent system refers to a method that applies low-density materials to obtain sufficient buoyancy, forms a gel layer, or changes its shape or structure [32].

Famotidine (FMT) is a histamine H_2_-receptor antagonist. It is widely prescribed to treat gastric and duodenal ulcers, Zollinger-Ellison syndrome, and reflux esophagitis. For the treatment of gastric and duodenal ulcers, the dose is 40 mg daily before bedtime for 4–8 weeks. The recommended dose for gastroesophageal reflux disease is 20 mg orally twice daily for 6–12 weeks. For short-term symptom relief, heartburn, or non-ulcer indigestion, the recommended dose is 10 mg twice a day. The initial treatment for Zollinger-Ellison syndrome is 20 mg every 6 h, which can be increased to 80 mg per day if necessary [34]. Due to its low bioavailability (45 ± 14%) and short biological half-life (2.5–3.5 h), FMT can cause diarrhea, dizziness, and headache [35]. Owing to these properties, research on sustained-release formulations of FMT has been actively conducted.

In this study, a low-density, partially filled internal structure gastro-floating tablet (GFT) was fabricated using SSE 3D printing technology. Nine GFTs were developed using combinations of three different hydroxypropyl methylcellulose (HPMC) concentrations and three different internal infill percentages. Their physicochemical characteristics, in vitro dissolution, floating abilities, and the release kinetics were also investigated. It is possible that this technique will eventually improve patient compliance and therapeutic effects while increasing FMT bioavailability. This study introduced a new formulation method of producing GFTs using 3D printing, which is highlighted as a future pharmaceutical manufacturing technology. To the best of our knowledge, this is the first work to parlay SSE 3D printing into developing sustained release gastro-floating famotidine tablets. Furthermore, this development is expected to provide information that can be the basis for future research.

## 2. Material and Methods

### 2.1. Materials

As shown in Table 1, FMT was purchased from the Tokyo Chemical Industry Co., (Tokyo, Japan). HPMC 2208 was donated by the Hanmi Pharmaceutical Co., (Hwasung, Republic of Korea). α-lactose monohydrate and microcrystalline cellulose 20–100 µm (MCC) were purchased from the Daejung Chemical Co., (Siheung, Republic of Korea). Polyvinylpyrrolidone K30 (PVP) was supplied by BASF SE. (Ludwigshafen, Germany). Distilled water used in the experiment is distilled water, and other reagents and solvents were of HPLC or analytical grade.

### 2.2. Preparation of the FMT-Loaded Paste

Before forming the FMT-loaded paste, HPMC (7%, *w*/*v*) gel was prepared, which is the base of the paste. Briefly, 15 mL of water was mixed with 15 mL of ethanol at a 1:1 ratio to prepare 30 mL of hydroalcoholic solvent. After heating the solvent to 70 °C, 2.1 g of HPMC with a viscosity grade of 4000 cps was added, and the solution was mixed for 30 min until complete dissolution. The prepared hydroalcoholic gel was sealed and stored for 24 h to allow air bubbles to escape and a homogeneous concentration to form. HPMC (5%, *w*/*v*) and HPMC (9% *w*/*v*) were also prepared using the same methods as described above.

Using a bench-top blender, FMT powder, lactose, MCC, and PVP K30 were used at the ratios shown in Table 2. The pre-adjusted hydroalcoholic HPMC gel was mixed with evenly mixed FMT and other excipients for 30 min until a uniform paste was observed without clumping.

Each syringe used in the 3D printer was filed with the prepared paste. To obtain uniform 3D printed tablets, an air bubble removal process was performed when filling the syringes. An empty syringe was connected to a filled syringe with a mixing tube to move the paste and remove air bubbles. The FMT-loaded paste, from which the air bubble was removed, was filled into the 3D printing syringe-type cavity. When air pressure was applied to the 3D printer, the filled paste was extruded through a 0.6 mm diameter nozzle.

### 2.3. Fabrication of 3DP-FMT Tablets Using SSE 3D Printing

The previously prepared paste was used to fabricate GFTs using a commercial extrusion-based printer, Dr. INVIVO 4D6 (Rokit Healthcare, Seoul, Republic of Korea). Tinkercad (Autodesk Inc., San Rafael, CA, USA) was used to design the 3DP-FMT, which have cylindrical dimensions of X = 10.0 mm, Y = 10.0 mm, and Z = 5.0 mm. The designed file was exported as a stereolithography (.stl) file to NewCreatorK version 1.57.70 (Rokit healthcare, Seoul, Republic of Korea). Tablets designed in the software program were prepared to compare dissolution profiles by setting different infill percentages (10%, 30%, and 50%). Other printer conditions were set as follows: nozzle size 20 G (0.60 mm), layer height (0.35 mm), print speed (5 mm/s), travel speed (5 mm/s), Infill overlap (15%), shell thickness (0.60 mm), printing temperature (35 °C). In the post-printing process, the 3DPs were dried in an oven at 50 °C for 24 h.

### 2.4. Analyzing the Physicochemical Characteristics of 3DP-FMT

#### 2.4.1. Determination of the Drug Contents of 3DP-FMT

Three 3DP-FMTs were finely powdered, 50 mg of each formulation was accurately measured, and 100 mL of a solvent containing methanol and 0.1% orthophosphoric acid at a 55:45 ratio was added to a transparent volumetric flask and shaken until completely dissolved. A drug content test was performed using the HPLC-UV method. The HPLC conditions were as follows: the HPLC apparatus was equipped with a Dionex UltiMate 3000 (Thermo Scientific, Bremen, Germany), separation was performed by an C18 (4.6 mm id, 250 mm, 5 μm) reverse phase column (Osaka Soda, Osaka, Japan), the mobile phase consisted of methanol and 0.1% orthophosphoric acid at a volume ratio of 55:45, the flow rate was set to 1.0 mL/min, and the detection wavelength was 265 nm.

#### 2.4.2. Calculating the Weight and Size Variations of 3DP-FMT

For each formulation, 20 tablets were randomly selected and accurately weighed on an electronic scale. The diameter and thickness of the tablets in the X- and Y-directions were measured using Vernier calipers and the average and deviation were identified.

#### 2.4.3. Determination of 3DP-FMT Strength

Tablets should have sufficient hardness to withstand certain forces. Six 3DP-FMTs were randomly selected for each formulation and their hardness was measured with a tablet hardness tester EH-01 (Electrolab, Mumbai, India). Hardness is expressed in N (Newtons).

#### 2.4.4. Determination of Tablet Friability

Twenty tablets of each formulation were randomly selected and placed on a sieve. A soft brush was used to remove dust or powder adhered to the surface of the tablet. The tablets were weighed precisely, placed in the drum of a friability tester TAR120 (ERWEKA; Heusenstamm, Germany), and rotated for 4 min at a constant speed of 25 rpm. The mass was measured again after the powder was removed from the tablet. The friability value was derived using the following equation by calculating the proportion of mass loss measured before and after the experiment. A maximum mean weight loss from the samples of not more than 1.0% is considered acceptable for most products.

Weight loss (%) = (W_1_ − W_2_)/W_1_ × 100

where, W_1_ = weight of tablets before tumbling (g)

W_2_ = weight of tablets after tumbling (g)

#### 2.4.5. Scanning Electron Microscopy (SEM)

The surfaces of FMT, HPMC, lactose, MCC, and PVP K30, the physical mixture (PM) of drugs and excipients used in the experiment, and the surface of 3DP-FMT were examined using a scanning electron microscope (SU-8220; Hitachi, Tokyo, Japan) at an accelerated voltage of 5.0 kV. Double-sided adhesive tape was used to attach the samples to a brass specimen holder, and an EmiTeck Sputter Coater K575 K (Quorum Technologies Ltd., West Sussex, UK) was used to coat the samples with platinum (6 nm/min) in a vacuum (0.8 Pa) for 4 min at 15 mA. This made the samples electrically conductive.

#### 2.4.6. Differential Scanning Calorimetry (DSC)

Thermal analysis was performed using a differential scanning calorimeter (DSC Q20; TA Instruments, Newcastle, DE, USA). The samples (approximately 5 mg) were weighed, sealed, and placed in an aluminum pan. Under a nitrogen gas flow of 50 mL/min, the samples were heated from 50 °C to 200 °C at a constant temperature change rate of 10 °C/min. The TA 2000 analysis program was used to analyze the data.

#### 2.4.7. Powder X-ray Diffraction (PXRD)

All powder samples were analyzed using a powder X-ray diffractometer (D/MAX-2500; Rigaku, Tokyo, Japan) equipped with a copper anode and operating with Cu K radiation (1.54178, 40 kV, and 40 mA). To obtain the diffraction patterns, samples were scanned in step scan model from 5° to 50° at room temperature with a step size of 0.05°/s.

#### 2.4.8. Fourier Transform Infrared Spectroscopy (FTIR)

All powder samples (FMT, HPMC, lactose, MCC, PVP K30, PM, and 3DP-FMT) were analyzed using a Fourier transform infrared (FTIR) spectrometer (Cary630 FTIR, Agilent Technologies, Santa Clara, CA, USA). FTIR spectroscopy revealed the formation and interactions by assessing the changes in the peak shape, peak positions, and intensities. The analyzed wavelength range was 4000–400 cm^−1^ with 16 scans and 2 cm^−1^ resolution.

### 2.5. In Vitro Dissolution and Floating Study

The floating study was carried out at 50 rpm in a transparent glass beaker with 500 mL of 0.1 N HCl (pH 1.2) solution. The tablets were placed in a beaker and allowed to sink for 5 s using a glass rod, after which the time to rise from the bottom of the beaker was measured by withdrawing the glass rod.

In vitro dissolution tests were performed using USP type II dissolution apparatus (DT 620; ERWEKA, Heusenstamm, Germany). The GFTs were placed in 900 mL of 0.1 N HCl solution and stirred at 50 rpm while being kept at 37 ± 0.5 °C. At predetermined intervals (0, 5, 10, 15, 30, 45, 60, 90, 120, 180, 240, 360, 480, and 600 min), samples (1 mL) were extracted, and an equal volume of new solution was instantly replenished to compensate for the loss during sampling. The collected samples were filtered through a 0.45 μm PTFE membrane syringe filter and analyzed using the HPLC apparatus described above. The dissolution test was repeated three times for each formulation, and the average and deviation were measured.

### 2.6. Dissolution Kinetics Studies

To explain the FMT release pattern of 3DP-FMT, several mathematical models (zero-order, first-order, Higuchi, and Korsmeyer-peppas) were employed. The dissolution data was analyzed statistically by comparing the dissolution profiles with mathematical models to estimate and characterize drug release from 3DP-FMT. When the correlation coefficient (R^2^) values of the above models were compared, the model with the highest correlation coefficient (R^2^) showed the best drug release pattern.

## 3. Results and Discussion

### 3.1. Preparation of FMT-Loaded Paste

The excipients used in this study were HPMC 2280, lactose, MCC, and PVP K30. HPMC 2280 is a hydrophilic polymer that forms a matrix and is used as a binder for controlled drug release. If only water is used as the solvent to dissolve the HPMC, excessive hydration of the HPMC surface leads the particles to expand, resulting in nozzle clogging or irregularly formed tablets because of the poor fluidity and high viscosity of the paste. Furthermore, tablets printed from over-expanded HPMC exhibit excessive shrinkage and deformation after drying. Therefore, a hydro-alcoholic gel prepared with a solvent mixed with purified water and ethanol was used to slow the hydration and expansion of the hydro-alcoholic gel, suppressing the excessive shrinkage of the printed tablets [18]. Lactose and MCC were used as fillers. MCC was used as a squeeze and pushing agent because it can control the fluidity of paste during the 3D printing process and reduce the deformation of printed tablets [36]. PVP K30 was used as a binder to increase the hardness of the tablets [37].

### 3.2. Fabrication of 3DP-FMT Tablets Using SSE 3D Printing

**Figure 1:**
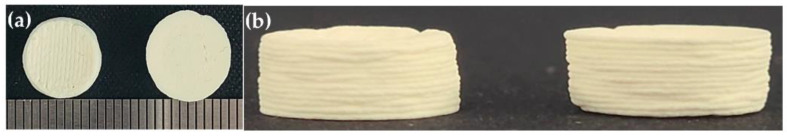
The appearance of F5. (**a**) The tablet’s top and bottom views. (**b**) Side view.

The 3D printing extrudability of the paste was evaluated. The characteristics of paste for successful printing in SSE printing technology are flowability, viscosity and formability [36,37]. Based on these properties, the optimal printing conditions were determined for each paste produced under different conditions. First, printing was performed by changing the composition ratio of HPMC from 5% (*w*/*w*) to 20% (*w*/*w*). When the HPMC concentration was low, the flowability and viscosity were good, so it could be easily extruded through the printer nozzle even when the pressure was low; however, the formability of the printed tablets after printing was poor. When the concentration of HPMC was high, the nozzle was frequently clogged because of its high viscosity and low flowability even under high pressure, resulting in inconsistent printing. Finally, the concentration of HPMC was increased from 10% (*w*/*w*) to 18% (*w*/*w*), and the printable range was confirmed. MCC also affected the printability of the paste. When the concentration of MCC in the paste was greater than 30% (*w*/*w*), the expansion or contraction of the printed tablet after printing was reduced. As shown in Figure 1b, layer-by-layer deposition consistently occurred. However, when the concentration of MCC was above 40% (*w*/*w*), nozzle clogging occurred frequently, resulting in non-uniform tablets. As a result, tablets were manufactured with three types of paste: MCC 30% (*w*/*w*), with three HPMC concentrations of 10%, 14%, and 18% (*w*/*w*).

**Figure 2:**
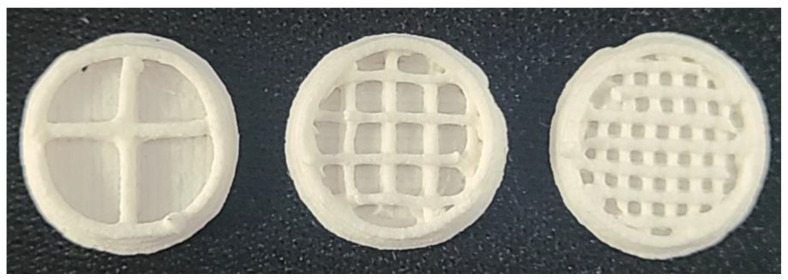
The section of F4–F6 with different infill percentages, 10%, 30%, and 50% from left to right.

**Figure 3:**
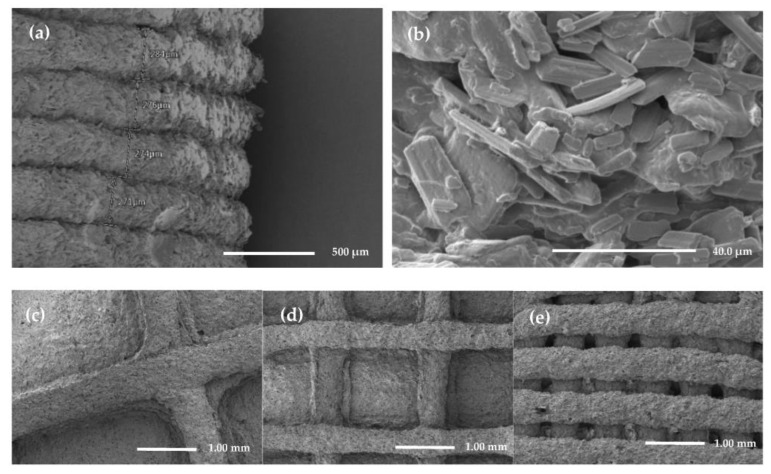
Scanning electron microscopy image of the printed tablets. (**a**,**b**) The side of printed tablet’s surface; (**c**–**e**) The cross-section of printed tablets with different infill percentages 10%, 30%, and 50%.

A total of nine types of tablets (F1–F9) shown in Table 3 were produced by setting the shape and size of the tablets to be the same as the three types of paste loaded with FMT and filling the inner cavity of the tablets with different infill percentages of 10%, 30%, and 50%. When the infill percentage was 0%, the inner side of the tablet was empty, and the top of the tablet collapsed after printing, making it difficult to maintain the tablet shape. However, shrinking of the inner structure occurred during the drying process in the case of tablets with an infill percentage of 70% or higher, resulting in the contraction of the bottom and top of the printed tablets. Therefore, tablets with three different infill percentages were produced with minimal shrinkage and deformation of the tablet shape. As shown in Figure 2 and Figure 3c–e, the inner space of the manufactured tablet was filled where the nozzle had passed, and the empty part at the other side was replaced with an air layer. As a result, the tablet retained as low density, allowing it to float.

### 3.3. Analyzing the Physicochemical Characteristics of 3DP-FMT

#### 3.3.1. Determination of the Drug Contents of 3DP-FMT

The drug contents of the tablets manufactured by SSE technology were analyzed using the HPLC-UV method. As shown in Table 2, the drug contents of the tablets made using pastes of HPMC 10% (*w*/*w*), HPMC 14% (*w*/*w*), and HPMC 18% (*w*/*w*) were 97.0 ± 0.2%, 96.9 ± 0.1%, and 97.2 ± 0.6%, respectively. This indicates that no significant drug losses occurred during paste preparation or tablet manufacturing, and the USP specifications for drug contents were satisfied.

#### 3.3.2. Geometry and Physical Properties of 3DP-FMT

Table 3 shows the diameter, thickness, weight, hardness, and friability of 3D-printed tablets. When compared to the tablet size designed with a diameter of 10.0 mm and a thickness of 5.0 mm, both diameter and thickness of the manufactured tablet had shrunk. However, it was confirmed that the diameter and thickness deviations of the manufactured tablets were reproducibly produced in the range of −0.33 mm to +0.33 mm for diameter, and −0.18 mm to +0.18 mm for thickness. Tablet shrinkage was observed (−1.42 mm to −0.74 mm from the set diameter, and −1.74 mm to −0.87 mm from the set thickness) under the same infill percentage conditions as the concentration of HPMC increased. Tablets manufactured with a HPMC composition ratio of 18% (*w*/*w*) shrank faster than those manufactured with other ratios of paste. This was caused by the evaporation of water and shrinkage of the expanded HPMC containing moisture during the drying process of the manufactured tablet [38].

The weight deviation of the same type of manufactured tablet followed the USP specification because it was manufactured with sufficient repeatability, with a deviation not exceeding 10% of the average weight. For weight variation of tablets manufactured with the same infill percentage, the tablets with higher HPMC ratios, particularly the 18% HPMC ratio (*w*/*w*), tended to be lighter. This is because, as the percentage of HPMC in the composition increased, more water-containing gel was formed, and the weight decreased due to water evaporation after drying.

Tablet hardness is an important evaluation criterion for solid tablets. Good tablets should have an appropriate hardness to meet the strength required for transportation and storage without significantly affecting the drug release mechanism. The required hardness value of a tablet varies depending on the tablet use, size, shape, or production process, and there is no clear standard for this. However, it is known to be satisfactory when the tablet hardness is 4 kg or more in normal cases [39]. In the conventional method, the hardness of tablets is controlled by the pressure of the machine. However, binders are used to control tablet hardness in the semisolid extrusion 3D printing process since there is no compression process. HPMC and PVP K30 were used as binders in this study. It was confirmed that the hardness of tablets manufactured with a paste with a high HPMC concentration and a hardness of 119.3–246.1 N satisfied the strength required for tablets.

The friability test is another USP specification for evaluating tablet resistance to chipping, capping, or abrasion during transportation and storage during the manufacturing process. The mass loss rate of 3D printed tablets was less than 0.21% (lower than 1.0%) which meets USP requirements.

#### 3.3.3. Scanning Electron Microscopy (SEM)

The surface and internal structures of 3D printed tablets were determined using scanning electron microscopy (Figure 3). Layer-by-layer deposition occurred consistently during the 3D printing process. The average thickness of each parallel layer was 276 μm, which was lower than the 350 μm layer height (the height of the nozzle that rose for printing the next layer after deposition) set in the printing setting. This demonstrated that the HPMC gel with water swelled during the printing process but contracted after drying. HPMC, a hydrophilic polymer, absorbed sufficient water by replacing the hydroxyl group in the original cellulose backbone and forming a new ether bond, thereby increasing the water solubility [40,41]. Substituted methoxy groups prevented hydrogen bond formation and increased solubility. This substituted methoxy group aggregated via hydrophobic intermolecular bonds, causing the gelation of HPMC [42]. As the concentration of HPMC increased, more methoxy groups interacted with water. The strength and viscosity of the gel naturally increased as water absorption and intermolecular interactions increased [43]. FMT and other excipients, such as MCC, were mixed into the formed gel to create a paste, which acted as a viscosity modifier and deformation resistance.

#### 3.3.4. Differential Scanning Calorimetry (DSC)

**Figure 4:**
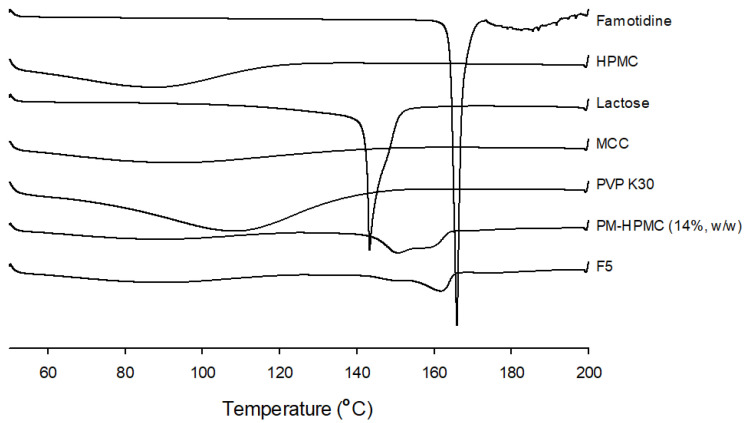
Differential scanning calorimetry images of famotidine, hydroxypropyl methylcellulose, lactose, microcrystalline cellulose, polyvinylpyrrolidone K30, the physical mixture, and the three-dimensional-printed tablet. HPMC, hydroxypropyl methylcellulose; MCC, microcrystalline cellulose; PVP, polyvinylpyrrolidone.

FMT powder, excipients (HPMC, lactose, MCC, and PVP K30), the PM, and the printed formulation were evaluated using differential scanning calorimetry (DSC). According to the DSC results shown Figure 4, FMT showed a strong endothermic peak at approximately 162–165 °C, corresponding to its melting point. HPMC, MCC, and PVP K30 showed a broad endothermic peak during the heating process, whereas lactose showed a strong endothermic peak at 143 °C. This peak was produced by the loss of water molecules [36,44]. The peaks of FMT and lactose shifted to a broad peak from 140 °C to 162 °C in the physical mixture result, and the endothermic peak of FMT shifted to a lower temperature in the 3D printed tablet. Complementary investigations were conducted to further understand the observed changes.

#### 3.3.5. Powder X-ray Diffraction (PXRD)

**Figure 5:**
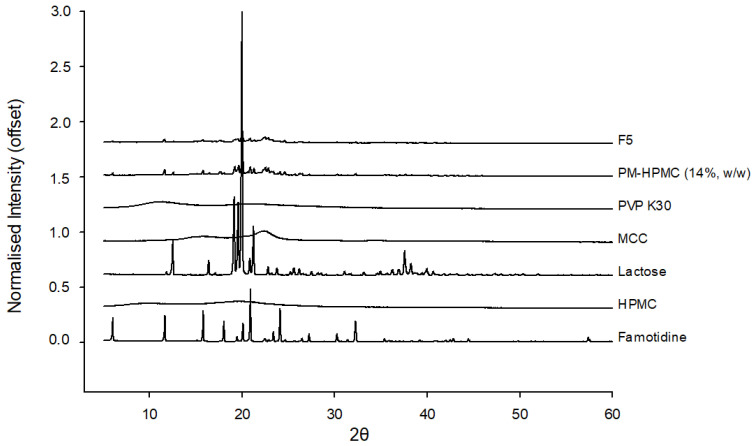
Powder X-ray diffraction images of famotidine, hydroxypropyl methylcellulose, lactose, microcrystalline cellulose, polyvinylpyrrolidone K30, the physical mixture, and the three-dimensional-printed tablet. HPMC, hydroxypropyl methylcellulose; MCC, microcrystalline cellulose; PVP, polyvinylpyrrolidone.

Figure 5 shows the X-ray diffraction analysis results of the raw materials, PM, and printed tablets. Sharp diffraction patterns of pure FMT could be observed at 11, 16, 19, and 21° (2θ diffraction angle), proving the intrinsic crystallinity of the FMT. Lactose showed sharp peaks, especially at 20°, and broad peaks were observed for other excipients. In the PM, the intrinsic diffraction pattern of FMT and excipients was maintained, but the intensity decreased. This can be explained by the dilution effect of pure crystalline components [45]. FMT diffraction patterns were also observed in 3D printed tablets, but their intensity was reduced further, and the lactose peak, which showed strong intensity at 20° disappeared. This indicated that FMT was partially dissolved in the paste created by dissolving lactose and hydrophilic excipients in a solvent, so that the crystalline and amorphous states coexisted in addition to the diluting effect of the excipient [36].

#### 3.3.6. Fourier Transform Infrared Spectroscopy (FTIR)

**Figure 6:**
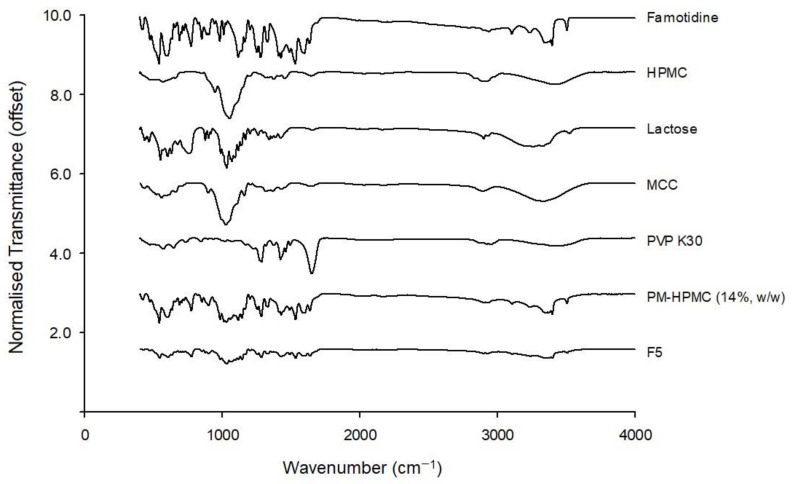
FTIR images of famotidine, HPMC, lactose, MCC, PVP K30, physical mixture, and 3D printed tablet.

Figure 6 shows the FTIR spectra results of FMT, excipients, the PM, and the 3D-printed tablets. The spectrum of pure famotidine showed trembling bands for the amine group (3505, 3400 and 3375 cm^−1^), stretching of C-H group (2936, 3104 and 3236 cm^−1^), C=N stretching (1638 cm^−1^), amine group bending (1600 cm^−1^), N=O (1534 and 1429 cm^−1^), and asymmetric stretching of sulfonyl group (1284 and 1147 cm^−1^). Similar spectrum has been reported previously [46]. The spectra of all components can be seen in the spectrum of the PM, indicating that no chemical interaction between FMT and excipients occurred during the mixing process. The 3D-printed tablets show the same pattern except for the 3100–3500 cm^−1^ range. The characteristic peaks of the above-mentioned part generally have an amine and a hydroxyl group, and the loss of this peak can be confirmed in the manufactured formulation. In the process of developing FMT-loaded paste, the components are dissolved or dispersed in a solvent, suggesting that the amine and hydroxyl groups of FMT interact to form a new hydrogen bond [47,48].

### 3.4. In Vitro Dissolution and Floating Study

**Figure 7:**
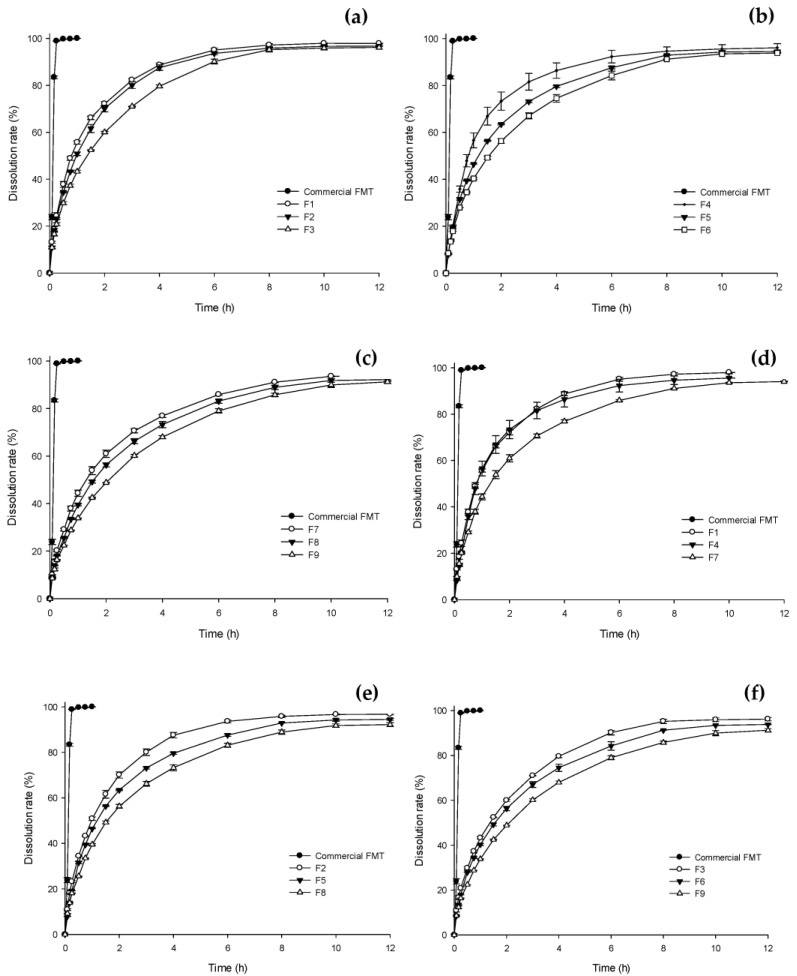
Drug dissolution profiles of three-dimensional printed tablets with different formulations. The release profiles were determined by the infill percentage at the same hydroxypropyl methylcellulose (HPMC) concentration; (**a**) HPMC 10% (*w*/*w*), (**b**) HPMC 14% (*w*/*w*), and (**c**) HPMC 18% (*w*/*w*). Additionally, the release profiles were determined by the HPMC concentration at the same infill percentage; (**d**) 10% infill percentage, (**e**) 30% infill percentage, and (**f**) 50% infill percentage. Each value represents the mean ± standard deviation (n = 3). HPMC, hydroxypropyl methylcellulose.

In vitro dissolution studies of nine HPMC-based formulations were performed for 12 h in 0.1 N HCl (pH 1.2), which represents the acidic gastric fluid for gastro-retentive systems [49,50]. Figure 7 shows the results of comparing the dissolution pattern difference according to the concentration of HPMC and the difference in infill percentage in 3D-printed tablets. Compared to commercial FMT tablets, all of the 3D-printed tablets were effectively manufactured as sustained-release floating tablets. The results shown Figure 7a–c shown the dissolution results by changing the infill percentage while fixing the HPMC composition ratio to 10%, 14%, and 18% (*w*/*w*), respectively. It was confirmed that the lower the infill percentages, the faster the dissolution rate for all the fabricated 3D-printed tablets. This result was caused by the porosity and cavity between the successively deposited layer at lower infill percentages. The high porosity allows for the rapid penetration of the solvent into the tablet core, leading to rapid release of FMT from the HPMC matrix. According to previous studies, tablets with high infill percentages are harder and encounter a stronger retarding force, which counters the effects of polymer dissolution and delays the drug release rate [50]. In particular, this difference was evident in the formulation with a 14% (*w*/*w*) HPMC concentration (Figure 7b). The dissolution results of tablets manufactured at 10% and 30% infill percentages were similar in the formulation with an HPMC composition ratio of 10% (*w*/*w*). This could be because HPMC 10% (*w*/*w*) does not provide sufficient dissolution delay with a large cavity in the tablet at a low infill percentage. The results shown in Figure 7d–f are comparisons of dissolution patterns by changing the HPMC composition ratio with the infill percentage fixed at 10%, 30%, and 50%. For all three types of infilled tablets, it was shown that the higher the HPMC composition ratio, the slower the dissolution rate. It has been reported that HPMC delays drug dissolution through two mechanisms: release from the hydrogel in printed tablets by forming a hydrophilic matrix, and erosion, which delays the water diffusion of FMT by erosion of the porous structure [51,52]. The difference in dissolution pattern according to the HPMC composition ratio showed the greatest difference when the infill percentage ratio was 30% (Figure 7e). However, in the formulation with an infill percentage of 10%, the dissolution patterns of the tablets manufactured with the HPMC composition ratio of 10% and 14% (*w*/*w*) was similar. If the infill percentage is 10% and the HPMC composition ratio does not exceed a certain level, it is because a large cavity in the tablet act as an obstacle to formation of a matrix that will delay the drug release rate.

**Figure 8:**
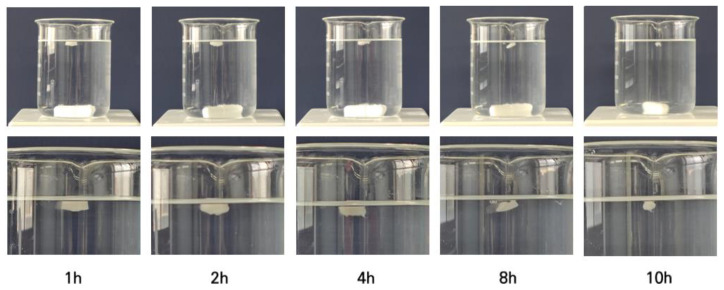
Images of 3D printed tablet floating in dissolution medium (0.1 N HCl solution) at room temperature.

The manufactured tablets were stirred in a 0.1 N HCl solution at 50 rpm to check their floating ability (Figure 8). All nine formulations floated up as soon as they were immersed in the beaker. The floating lag time was determined to be 0 s, and they floated for more than 10 h. By controlling the infill percentage, the formulations could have a lower density because the inner space of the tablet was not completely filled, and the large amount of air in the cavity provided buoyancy and allowed them to float longer [19].

### 3.5. Dissolution Kinetics Studies

Mathematical modeling is required to investigate in vitro/in vivo drug release kinetics and predict drug release profiles in order to determine optimal dosage forms [50]. The drug release pattern was applied to the zero order, first order, Higuchi, and Korsmeyer-Peppas models to analyze the kinetics of the FMT floating tablets (Table 4).

(1) $$\frac{M_{t}}{M_{\infty }}=kt^{n}$$

This equation is a comprehensive semi-empirical equation that describes drug release from polymer systems when the drug release pattern is unknown or when more than one type of mechanism is involved. *M_t_* is the amount of drug dissolved at time *t* and *M_∞_* is the total amount of drug contained in the formulation before the start of dissolution. *k* is a constant that integrates the structure and geometry of the device, and *n* is the release exponent related to the drug release mechanism [53].

In general, the first 60% of the drug release curve is used for statistical analysis [54], and the mechanism by which the drug is released from the matrix system is proposed based on the release parameter (R^2^) and the release exponent (n). For cylindrical tablets (Case I), n ≤ 0.45 is explained by Fickian diffusion, in which drug molecules are released by the diffusion process. When n = 0.89, this model is non-Fickian (Case II transport), and the drug release corresponds to zero-order release. Drug release primarily involves the expansion or relaxation of polymer chains in a drug-loaded matrix system. If 0.45 < n < 0.89, the model indicates non-Fickian or anomalous transport, and trug release occurs as a diffusion and swelling mechanisms. If n > 0.89, it represents a super case II transport model in which drug release is caused by the sorption process, tension, and breaking of the polymer chain. All nine formulations best fit the Korsmeyer-Peppas model, and the n value (as in Equation (1)) ranged from 0.45 to 0.89. This means that the drug release proceeds via diffusion and swelling mechanisms in the HPMC matrix in the formulations manufactured for GFTs.

## 4. Conclusions

3D-printed GFTs were successfully fabricated using FMT-loaded HPMC-based pastes. The manufactured hollow 3D tablets with various infill percentages and HPMC concentrations demonstrated sufficient buoyancy for approximately 10 h, presenting a novel method for fabricating controlled-release intragastric floating drug delivery systems. The critical parameters for in vitro drug release were discovered for different infill percentages (10%, 30%, and 50%) and HPMC concentrations (10%, 14%, and 18% *w*/*w*). All nine drug release profiles fit the Korsmeyer–Peppas release kinetics, which included both swelling and diffusion mechanisms. Overall, this study revealed that SSE 3D printing technologies could be an efficient, effective, and cost-effective alternative to traditional pharmaceutical manufacturing for developing gastro-retentive dosage forms with better controlled-release rates. However, additional stability and in vitro/in vivo correlation tests of 3DP floating formulations are required. Despite substantial advances, 3D printing technology is still in early stages of development. Further innovations in quality, higher printing resolution, and appropriate regulatory systems will enable this promising technology to become a practical method for commercial manufacture.

## Figures and Tables

**Table 1:** Materials and manufacturing company.

Materials	Manufacturing Company
Hydroxypropyl methylcellulose (HPMC 2208)	Hanmi Pharmaceutical Co., (Hwasung, Republic of Korea)
α-lactose monohydrate	Daejung Chemical Co., (Siheung, Republic of Korea)
MCC (microcrystalline cellulose 20–100 µm)	Daejung Chemical Co., (Siheung, Republic of Korea)
PVP (polyvinylpyrrolidone) K30	BASF SE. (Ludwigshafen, Germany)
Famotidine	Tokyo Chemical Industry Co., (Tokyo, Japan)

**Table 2:** The percentage composition of various ingredients in famotidine loaded paste at different hydroxypropyl methylcellulose concentrations.

Formulation Name	Composition (*w*/*w*)				
	Famotidine (%)	HPMC (%)	Lactose (%)	MCC (%)	PVP K30 (%)
HPMC (10%, *w*/*w*)	25	10	20	40	5
HPMC (14%, *w*/*w*)	25	14	16	40	5
HPMC (18%, *w*/*w*)	25	18	12	40	5

HPMC, hydroxypropyl methylcellulose; MCC, microcrystalline cellulose; PVP, polyvinylpyrrolidone.

**Table 3:** Physical properties of 3D-printed tablets. Each value represents the mean ± standard deviation (n = 20). Except hardness (n = 6).

Formulation Name	Geometry and Properties				
	Diameter (mm)	Thickness (mm)	Weight (mg)	Hardness (N)	Friability (%)
F1 (HPMC (10%, *w*/*w*)-Infill 10%)	9.04 ± 0.29	3.85 ± 0.10	129.9 ± 7.3	121.5 ± 2.2	0.13
F2 (HPMC (10%, *w*/*w*)-infill 30%)	9.07 ± 0.26	3.87 ± 0.06	151.1 ± 8.9	123.5 ± 4.3	0.15
F3 (HPMC (10%, *w*/*w*)-infill 50%)	9.26 ± 0.33	4.13 ± 0.18	193.3 ± 9.3	127.3 ± 4.8	0.10
F4 (HPMC (14%, *w*/*w*)-infill 10%)	9.05 ± 0.08	3.76 ± 0.09	115.5 ± 2.9	119.3 ± 4.4	0.13
F5 (HPMC (14%, *w*/*w*)-infill 30%)	9.02 ± 0.13	3.87 ± 0.11	137.5 ± 4.3	126.8 ± 2.3	0.21
F6 (HPMC (14%, *w*/*w*)-infill 50%)	9.14 ± 0.09	3.86 ± 0.14	165.9 ± 4.2	147.5 ± 5.2	0.15
F7 (HPMC (18%, *w*/*w*)-infill 10%)	8.82 ± 0.12	3.30 ± 0.07	111.9 ± 4.7	124.8 ± 2.1	0.17
F8 (HPMC (18%, *w*/*w*)-infill 30%)	8.58 ± 0.13	3.26 ± 0.08	127.1 ± 7.9	170.8 ± 3.6	0.14
F9 (HPMC (18%, *w*/*w*)-infill 50%)	8.62 ± 0.15	3.38 ± 0.09	161.9 ± 4.6	246.1 ± 5.6	0.11

**Table 4:** Fitting parameters of famotidine release from 3D-printed tablets.

Formulation Name	Zero Order (R^2^)	First Order (R^2^)	Higuchi (R^2^)	Korsmeyer-Peppas (R^2^)	n Value
F1	0.8112	0.9603	0.9912	0.9950	0.555
F2	0.8197	0.9504	0.9933	0.9981	0.558
F3	0.7789	0.9067	0.9984	0.9993	0.526
F4	0.8980	0.9924	0.9606	0.9876	0.654
F5	0.8700	0.9680	0.9777	0.9934	0.612
F6	0.8358	0.9355	0.9898	0.9971	0.573
F7	0.8286	0.9381	0.9926	0.9985	0.565
F8	0.8435	0.9345	0.9914	0.9994	0.576
F9	0.8197	0.9048	0.9950	0.9993	0.554

## Data Availability

The data presented in this study are available in this article.
